# Poor Illness Perceptions Are a Risk Factor for Depressive and Anxious Symptomatology in Fibromyalgia Syndrome: A Longitudinal Cohort Study

**DOI:** 10.3389/fpsyt.2017.00217

**Published:** 2017-11-01

**Authors:** Eric W. de Heer, Johanna E. Vriezekolk, Christina M. van der Feltz-Cornelis

**Affiliations:** ^1^Centre of Excellence for Body, Mind and Health, GGz Breburg, Tilburg, Netherlands; ^2^Tranzo Department, Tilburg School of Behavioural and Social Sciences, Tilburg University, Tilburg, Netherlands; ^3^Department of Rheumatology, Sint Maartenskliniek, Nijmegen, Netherlands

**Keywords:** pain, coping, illness perceptions, depression, anxiety, fibromyalgia, longitudinal

## Abstract

**Background:**

Patients with widespread pain, such as in fibromyalgia, are vulnerable for depression and anxiety, which composes a relevant public health problem. Identifying risk factors for the onset of depression and anxiety is therefore warranted. Objective of this study was to determine whether severe pain, maladaptive coping, and poor illness perceptions are associated with depressive and anxious symptomatology in fibromyalgia.

**Method:**

Consecutive patients referred to an outpatient clinic completed sets of physical and psychological questionnaires at baseline and at 18-month follow-up. A total of 452 patients with fibromyalgia syndrome (FMS) were eligible for inclusion, and subsequently, 280 patients returned the baseline questionnaire. Depressive and anxious symptomatology was measured with the Hospital Anxiety and Depression Scale. To measure pain severity, coping style, and illness perceptions, the Fibromyalgia Impact Questionnaire, Pain Coping Inventory, and the Illness Perception Questionnaire-Revised (IPQ-R) were used, respectively. Multivariable logistic regression analyses, bootstrapping and calibration, were performed to examine the association of pain severity, pain coping, and illness perception with depressive and anxiety symptoms at follow-up, adjusted for sociodemographic variables. Initial level of depressive and anxiety symptoms was selected as covariates.

**Results:**

Mean age was 42.6 years and 95.4% were female. At 18-month follow-up, 68 (of the 195) patients were depressed and 80 (of the 197) were anxious. Only the IPQ-R subscale “emotional representations” showed a significant positive association with depressive symptoms at follow-up (OR = 1.10), next to the initial level of depressive symptoms (OR = 1.30). In case of anxiety, only the IPQ-R subscale “treatment control” showed a significant negative association with anxiety symptoms at follow-up (OR = 0.87), next to the initial level of anxiety symptoms (OR = 1.45).

**Conclusion:**

Our data suggest that not pain severity or maladaptive coping, but poor illness perceptions are important in elevated depressive and anxious symptomatology. Patients with fibromyalgia who think their illness negatively affects their mental well-being are at increased risk for more depressive symptoms, and those who think treatment of their illness will not be effective are at increased risk for more anxiety symptoms. Strengthening illness beliefs and reducing catastrophic thinking, therefore, seem crucial factors in the treatment of patients with FMS.

## Introduction

Fibromyalgia syndrome (FMS) is a medical diagnosis of unknown etiology mainly characterized by chronic and widespread pain (1). Other symptoms include fatigue, gastrointestinal symptoms, and joint stiffness (1–3). Furthermore, a growing body of evidence shows a comorbidity of FMS with psychological symptoms, particularly with depression and anxiety (2–9): lifetime prevalence of depression and anxiety in patients with FMS go up to 70 and 60%, respectively (8). Widespread pain and mental disorders compose a relevant public health problem due to the high economic and societal burden (10–13). A better understanding of risk factors for the onset of depressive and anxious symptomatology in FMS might, therefore, be instrumental in identifying strategies for prevention and early interventions, such as those recommended by the European League Against Rheumatism (EULAR) (14, 15). Risk factors for the onset of depression and anxiety have been studied longitudinally, and include pain, a negative perception of health, dysfunctional coping strategies, personality traits (e.g., neuroticism), female gender, and lower education (10, 16). This study will focus on those factors that have shown to be associated with an increased risk of depression and anxiety in chronic pain patients, and that might be easily modifiable in treatment: severity of pain (10, 17–20), coping strategy (21–23), and illness perceptions (24–26). To date, longitudinal studies exploring the contribution of all these risk factors in subsequent depressive and anxious symptomatology in patients with FMS in one study are lacking.

Widespread pain is characteristic for FMS (1) and thorough research exists exploring the association of widespread pain and severity of pain with depressive and anxiety disorders (10, 17–20). In several cross-sectional studies, among which a review and a large survey among 17 countries, multiple pain locations were more strongly associated with depression and anxiety when compared with one pain location and no pain (10, 17, 18). In two longitudinal studies, every additional pain location increased the risk of a new onset and recurrence of a depressive and/or anxiety disorder with 29 and 7%, respectively (19, 20). Moreover, for every increase in the severity of pain the risk increased with 57% for new onset and 11% for recurrence of depression and/or anxiety (19, 20).

Another risk factor associated with depression and anxiety is coping style. Patients with FMS tend to cope passively, such as catastrophizing (27, 28). In patients with FMS, pain is indirectly associated with depression through its relationship with a passive coping style (21). A passive coping style, in turn, is significantly associated with depression (27). Furthermore, in studies among patients with other rheumatic diseases, passive coping strategies, including withdrawal and worrying or catastrophizing, were also associated with psychological distress (22, 23).

A maladaptive coping style is associated with poor illness perceptions (29), that in turn are associated with depressive and anxiety symptoms (24–26, 29, 30). In patients with chronic widespread pain, negative beliefs about their illness, such as that the illness would affect their emotional well-being, were found to be associated with an increase of depressive and anxious symptomatology (24, 25). Also, patients with rheumatoid arthritis who had negative beliefs about the consequences of their illness were more depressed over time (26).

Patients with FMS are characterized by pain in multiple locations, or widespread pain. This makes them vulnerable for depression and anxiety. However, it is unknown whether other characteristics are associated with the onset of depression and anxiety. Cross-sectional studies suggest that more severe pain, a maladaptive (passive) coping style, and negative illness perceptions are related to depression and anxiety in patients with (chronic) pain and in patients with rheumatic diseases (17, 18, 21–25). FMS is a rheumatic disease, characterized by chronic pain, which makes it likely that these factors would also play a role in the onset and persistence of depressive and anxious symptomatology in this population. However, these factors have not been studied in concert and longitudinally in FMS before. Therefore, a study exploring this association in a longitudinal design in FMS is warranted. The aim of this study is to examine pain, pain-related coping, and illness perception as possible risk factors for depressive and anxious symptomatology in patients with FMS. We hypothesize that more pain, passive pain-related coping and poor illness perceptions at baseline are associated with more depressive and anxiety symptoms in FMS.

## Materials and Methods

### Study Design

This study used data from an observational, prospective cohort study, with follow-up every 6 months for up to 18 months. Not all questionnaires of interest were used at every follow-up measurement, and, therefore, the baseline and 18-month data were used for this study.

### Setting

Data were collected of newly referred consecutive patients to the Sint Maartenskliniek rheumatology outpatient clinic, location Nijmegen and Woerden, the Netherlands, between December 2011 and November 2014.

### Ethics Statement

This study was carried out in accordance with the recommendations of the local medical ethical board with written informed consent from all subjects. All subjects gave written informed consent in accordance with the Declaration of Helsinki. The Institutional Review Board of the University Medical Centre, Nijmegen, approved the study (protocol number: 2011/271).

### Participants

**Figure 1:**
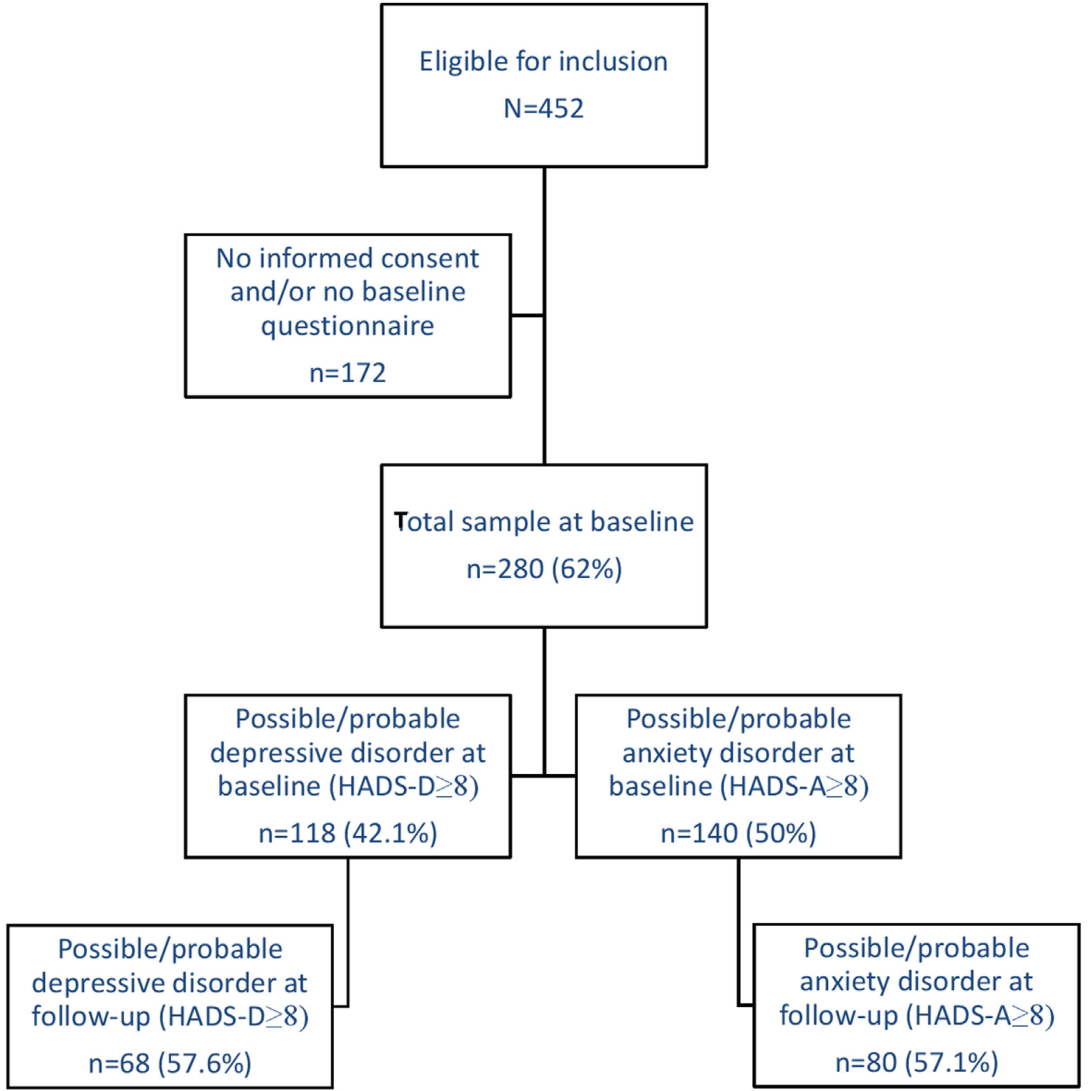
Flowchart of the inclusion procedure.

Patients were included in the cohort after being classified as having FMS by certified rheumatologists, who followed the ACR guidelines (31, 32) as a diagnostic aid. Other inclusion criteria were being 18 years or older and to be able to read and write in the Dutch language. There were no exclusion criteria next to these inclusion criteria. A total of 452 patients with FMS were eligible for inclusion, 311 gave informed consent (68.8%), and subsequently, 280 patients returned the baseline questionnaire (90.0%). Most frequent dropout reasons were lack of energy or concentration, and lack of time. Several patients did not specify a reason. For this study, all 280 participants were included. See Figure 1 for a flowchart.

### Variables

#### Dependent Variables

The primary outcomes were the presence of depressive symptoms (yes/no) and the presence of anxiety symptoms (yes/no). Anxiety and depressive symptomology were assessed using the self-rated, 14-item Hospital Anxiety and Depression Scale (HADS) (33), which scores the severity of depressive (HADS-D) and anxiety (HADS-A) symptoms, both subscales ranging from 0 to 21. This measure was developed specifically for medically ill populations and excludes bodily symptoms such as sleep disturbance, fatigue, and pain that may be directly attributable to the physical illness (33). The HADS has been widely used in people with medical illnesses, and its factor structure has been confirmed (34–36). The presence of depressive and anxiety symptoms was defined using the cutoff scores of ≥8 for “possible/probable” depressive or anxiety disorder (33). The cutoff score of 8 or more for both subscales has shown good predictive value for depressive and anxiety disorders, in somatic, psychiatric, and primary care patients, as well as in the general population (37). Both subscales of the HADS have a good internal consistency (Cronbach’s alpha for HADS-D ranges from 0.67 to 0.90; Cronbach’s alpha for HADS-A ranges from 0.68 to 0.93) (37).

#### Predictor Variables

##### Pain

Pain was assessed using the pain severity subscale of the Dutch translation of the Fibromyalgia Impact Questionnaire (FIQ) (38, 39), ranging from 0 (no pain) to 10 (severe pain). Reliability and validity of the FIQ are good (38).

##### Pain Coping

Pain coping was measured using the Pain Coping Inventory (PCI) (40). The PCI consists of six scales (33 items) measuring cognitive and behavioral pain-coping strategies that represent two higher order, active, and passive pain-coping dimensions. Items are rated according to a 4-point Likert scale ranging from 1 (hardly ever) to 4 (very often) in terms of frequency with which strategies are applied when dealing with pain. Active pain-coping strategies reflect three cognitive-behavioral strategies, measuring patient’s efforts to distract themselves from pain (distraction, five items), to reinterpret and transform the pain (pain transformation, four items), and to function despite pain (reducing demands, three items). Passive pain coping reflects three cognitive-behavioral strategies, assessing behavioral tendencies to restrict functioning (resting, five items), to avoid environmental stimuli (retreating, seven items), and catastrophic cognitions about the pain (worrying, nine items). A composite score of the active and passive coping dimensions can be calculated by taking the mean of the three active and passive coping strategies. Confirmatory construct and criterion validity of the scales and the second-order structure with respect to active and passive pain-coping strategies were supported (40, 41).

##### Illness Perception

Participants completed the Revised version of the Illness Perception Questionnaire (IPQ-R) (42), which assesses patients’ perception of their illness. The following seven dimensions of the IPQ-R were included: Timeline (e.g., “My illness will last a short time”), Timeline cyclical (e.g., “My symptoms come and go in cycles”), Consequences (e.g., “My illness is a serious condition”), Personal control (e.g., “Nothing I do will affect my illness”), Treatment control (e.g., “My treatment will be effective in curing my illness”), Illness coherence (e.g., “I do not understand my illness”), and Emotional representations (e.g., “My illness makes me feel angry”). The IPQ-R is a reliable and well-validated self-report questionnaire (42–44).

##### Covariates

Initial levels of depressive and anxiety symptoms play an important role in subsequent depressive and anxious symptomatology (20, 45). Therefore, baseline HADS-D score (initial level of depressive symptoms) and baseline HADS-A score (initial level of anxiety symptoms) were inserted in the statistical models as covariates.

#### Sociodemographic Variables

Assessed sociodemographic variables were as follows: sex, age, partner status, and education level (primary/lower secondary and higher secondary/professional).

### Statistical Methods

Descriptive statistics were performed to describe the sample at baseline, regarding sociodemographic variables, pain severity, pain coping, illness perceptions, and depressive and anxiety symptoms. To examine which variables predicted depressive (*n* = 68) and anxiety symptoms (*n* = 80) at 18-month follow-up, the following steps were performed: (1) selection of potential variables: univariate logistic regression analyses were performed, with *p* < 0.05 as a selection criterion for potential predictive variables; (2) multivariable logistic regression analyses using backward selection (stopping rule *p* < 0.2); (3) internal validation with a bootstrap procedure (500 samples) to estimate the amount of over-fit; and (4) through calibration, the slope value was calculated and used to correct and shrink the regression coefficients, the percentage of explained variance (*R*^2^), and the *c*-index. Initial level of depressive symptoms and initial level of anxiety symptoms were inserted in the models as covariates, and their association with the outcome variables was shown when significant. The models’ performance was assessed by the percentage of variance; the agreement between the predicted probabilities of the outcome and the observed probabilities in the original data (*p* > 0.05; i.e., Hosmer–Lemeshow test); and by the models’ discriminative ability [as reflected by the *c*-index that equals the area under the receiver operating characteristic curve (AUC)].

## Results

Table 1 shows the baseline characteristics of the 280 persons in this cohort. Mean age of the study sample was 42.6 years, and 95.4% were female. Mean pain severity was 6.75 on a 10-point Likert scale. Of the participants, 118 (42.1%) had a possible or probable depressive disorder, and 140 (50%) had a possible or probable anxiety disorder at baseline.

**Table 1:** Sociodemographic variables, pain severity, pain-coping strategies, and illness perceptions of the fibromyalgia cohort at baseline (*N* = 280).

Sex (*N*, %)	Male	13 (4.6)
	Female	267 (95.4)

Age (mean, SD)		42.61 (11.83)

Education level (*N*, %)	Low	122 (44)
	Middle	95 (34)
	High	55 (20)

Marital status (*N*, %)	Without partner	59 (21.1)
	With partner	218 (77.9)

Pain severity (mean, SD)		6.75 (2.09)
Pain-coping strategies (mean, SD)	Passive	43.83 (7.97)
	Active	29.16 (4.93)

Illness perceptions (mean, SD)	Timeline	23.71 (4.26)
	Consequences	19.86 (4.26)
	Personal control	20.34 (3.66)
	Treatment control	17.00 (2.88)
	Illness coherence	15.00 (4.09)
	Timeline cyclical	14.69 (3.27)
	Emotional representations	15.76 (4.74)

HADS depression (*N*, %)	Normal	162 (57.9)
	Possible/probable depression	118 (42.1)

HADS anxiety (*N*, %)	Normal	139 (50.0)
	Possible/probable anxiety	140 (50.0)

*HADS, Hospital Anxiety and Depression Scale*.

Table 2 shows the univariate associations between all predictor variables and the covariates, with depressive and anxious symptomatology at 18-month follow-up (*n* = 68 for depressive symptoms; *n* = 80 for anxiety symptoms). Regarding the depression outcome, a passive coping style (OR = 2.92), and the IPQ subscales “timeline” (OR = 1.09), “consequences” (OR = 1.22), “personal control” (OR = 0.89), and “emotional representations” (OR = 1.19) had a significant univariate association with a possible/probable depressive disorder at 18-month follow-up. The covariates’ initial level of depressive symptoms and initial level of anxiety symptoms also had a significant univariate association with a possible/probable depressive disorder at 18-month follow-up (OR = 1.37 and OR = 1.21, respectively). After logistic regression with backward selection (Table 3), the IPQ subscale “emotional representations” (OR = 1.10) was significantly associated with possible/probable depressive disorder at 18-month follow-up, next to initial level of depressive symptoms (OR = 1.30). Thus, subjects who respond emotionally regarding their illness and have more initial depressive symptoms at baseline are at increased risk for a possible or probable depressive disorder at follow-up. Together, these variables in the regression model accounted for 30% of the variance of the depression score at follow-up. The overall fit of the derived model was good (χ^2^ = 13.85, *p* = 0.09).

**Table 2:** Univariate logistic regression analyses of sociodemographics, pain severity, coping strategies, and illness perceptions, for depressive and anxiety symptoms.

	HADS depression (*n* = 68)	HADS anxiety (*n* = 80)
	
	OR (95% CI)	OR (95% CI)
**Sociodemographics**
Sex	1.06 (0.26–4.36)	1.39 (0.34–5.74)
Age	1.00 (0.98–1.03)	0.98 (0.96–1.01)
Having a partner	0.84 (0.41–1.70)	0.75 (0.38–1.49)
Higher educational level	0.61 (0.30–1.24)	0.62 (0.32–1.21)
Pain severity	1.15 (0.99–1.33)	1.13 (0.98–1.30)
**Coping strategy**		
Active coping	0.54 (0.26–1.13)	0.60 (0.30–1.20)
Passive coping	2.92 (1.27–6.73)*	2.80 (1.23–6.35)*
**Illness perceptions**		
Timeline	1.09 (1.01–1.17)*	1.06 (0.99–1.14)
Timeline cyclical	0.93 (0.85–1.02)	1.03 (0.94–1.12)
Consequences	1.22 (1.12–1.33)*	1.17 (1.08–1.26)*
Personal control	0.89 (0.81–0.97)*	0.94 (0.86–1.02)
Treatment control	0.91 (0.81–1.01)	0.90 (0.81–0.99)*
Illness coherence	0.96 (0.89–1.03)	0.95 (0.89–1.02)
Emotional representations	1.19 (1.10–1.28)*	1.19 (1.10–1.28)*
**Covariates**		
HADS baseline depression score	1.37 (1.24–1.52)*	1.21 (1.11–1.32)*
HADS baseline anxiety score	1.21 (1.12–1.32)*	1.44 (1.30–1.61)*

*HADS, Hospital Anxiety and Depression Scale*.

*Significant at the 0.05 level.

**Table 3:** Multivariable logistic model predicting depressive and anxiety symptoms at 18-month follow-up.

	HADS depression (*n* = 68)		HADS anxiety (*n* = 80)	
	OR	*p*-Value	OR	*p*-Value
HADS baseline depression score	1.30	<0.0001		
HADS baseline anxiety score			1.45	<0.0001
Treatment control			0.87	0.04
Emotional representations	1.10	0.03		

**Model performance**	**Model**	**Corrected**	**Model**	**Corrected**

Explained variance (Nagelkerke *R*^2^)	0.35	0.30	0.44	0.40
*c*-Index	0.82	0.80	0.85	0.83

**Calibration**				
Hosmer and Lemeshow	χ^2^ = 13.85		χ^2^ = 13.05	
Slope value	0.89		0.90	

*HADS, Hospital Anxiety and Depression Scale*.

Regarding anxiety outcome, a passive coping style (OR = 2.80), and the IPQ subscales “consequences” (OR = 1.17), “treatment control” (OR = 0.90), and “emotional representations” (OR = 1.19) had a significant univariate association with a possible/probable anxiety disorder at 18-month follow-up (Table 2). The covariates’ initial level of depressive symptoms and initial level of anxiety symptoms also had a significant univariate association with a possible/probable anxiety disorder at 18-month follow-up (OR = 1.21 and OR = 1.44, respectively). After logistic regression with backward selection, treatment control (OR = 0.87) was significantly associated with possible/probable anxiety disorder at 18-month follow-up, next to initial level of anxiety symptoms (OR = 1.45) (Table 3). Thus, subjects who think that their treatment is not effective regarding their illness and have more anxiety symptoms at baseline are at increased risk for a possible or probable anxiety disorder. Together, these variables in the regression model accounted for 40% of the variance of the anxiety score at follow-up. The overall fit of the derived model was good (χ^2^ = 13.05, *p* = 0.11).

## Discussion

To the authors’ knowledge, this is the first study to document on pain, illness perception, and coping style as risk factors for depressive and anxious symptomatology in patients with FMS. The results here indicate that patients with FMS who believe their illness will have a negative effect on their mental well-being, who also have elevated levels of depressive symptoms, are more likely to be depressed on subsequent occasions. Furthermore, patients with FMS who think that treatment of their illness will not be effective and who also have elevated levels of anxiety symptoms are more likely to be anxious on subsequent occasions. Our hypothesis that poor illness perceptions are a risk factor for depressive and anxious symptomatology is therefore confirmed.

Only illness perceptions and initial level of affective symptoms were found to play a significant role in depressive and anxious symptomatology, suggesting that these factors are possibly more important than other well-known risk factors, such as pain, which is contrary to what we expected. These findings are consistent with previous research in similar populations. For example, patients with chronic (widespread) pain who had a strong belief that their illness would affect their mental well-being were more likely to report depressive and anxiety symptoms (24, 25), whereas pain intensity did not show such an association (24). In patients with rheumatoid arthritis, the patients’ beliefs and emotional responses to their illness were key factors explaining the association between pain and depression (46). Furthermore, in two large longitudinal studies, the association between pain and new onset and recurrence of depression and anxiety were mediated by (subthreshold) depressive symptoms (19, 20). This could explain our finding that not pain severity, but negative cognitions about the illness and the initial level of affective symptoms are more important as risk factors in the development of subsequent depressive and anxious symptomatology. Neurobiological abnormalities might also play a role, which persists in patients who have experienced strong negative emotions, possibly through negative biases in the processing of emotional information (47). On the other hand, pain and emotion share neuronal networks, and pain, one of the main characteristics of FMS, might cause a dysregulation in the neuronal network associated with emotion (48). Thus, patients with FMS who experience negative affective symptoms and negative perceptions regarding their illness might, therefore, have developed neurobiological abnormalities, making them vulnerable for subsequent depressive and anxious symptomatology.

Contrary to what we expected, a passive coping style was not a significant risk factor for depressive and anxiety symptoms, in contrast to a previous study in patients with FMS (21). However, they used another construct of passive coping, emphasizing on emotion-focused strategies, whereas passive coping in this study was mostly characterized by avoidant strategies. In a study in patients with systemic sclerosis, a rheumatic disease, emotion-focused coping had a strong association with depressive symptoms, whereas avoidant focused coping did not (22). Furthermore, in patients with chronic heart failure, an avoidant coping style was indirectly associated with depression and anxiety through poor illness perceptions (29), which might explain our findings. This might suggest that emotional components of coping with an illness are more important than other maladaptive forms of coping. In our study, passive coping consisted of three cognitive-behavioral strategies, one of them being catastrophizing or worrying, which is an emotional component also. Therefore, future studies should explore the subcategories of passive coping to explore whether the emotional components of coping are indeed more important than other components.

Strengths of this study are the longitudinal design and the large sample size. However, several limitations need to be addressed. The sample of this study consisted of patients with FMS, which limits the generalizability to other populations. However, comparable results were found in other rheumatologic populations (22, 24, 25, 46). This might suggest that our results are generalizable to populations characterized by a chronic disease. In this study, the HADS, which was developed specifically for medically ill populations, was used to measure depressive and anxious symptomatology. Although this instrument has good internal consistency and can be used as case finding for depressive and anxiety disorders (37), and is widely used in rheumatologic populations, no inferences could be made whether patients met criteria for a depressive or anxiety disorder. For such purposes, diagnostic (semi)structured interviews need to be used, such as the Mini-International Neuropsychiatric Interview (49) or Composite International Diagnostic Interview (50). In addition, we used a single self-reported questionnaire (FIQ) to measure pain severity, which prevents us to make inferences of other aspects of functioning and well-being, such as quality of life. Besides, it is possible that factors not studied here might play a crucial role in depressive and anxious symptomatology in patients with FMS. Received treatment, whether it is physical (51–54), psychological, or pharmacological (55), can influence both depressive as anxiety symptoms. Patients in this study received advice for treatment, based on the recommendations of the EULAR (14, 15), after the baseline measurement. Treatment advice could be referral (back) to the general practitioner, physiotherapist, psychologist, or a multimodal rehabilitation program. Unfortunately, no information was available whether this advice was followed, or if another treatment was received other than advised. We were, therefore, unable to make any inferences on the effect of treatment on depression and anxiety. Other factors that might influence depressive and anxious symptomatology are medication use, comorbidity with other chronic diseases (56), and perceived social support (57). Unfortunately, due to the limited sample size at follow-up (68 patients for depressive symptoms and 80 for anxiety symptoms), it was not possible to include more factors in the statistical models. Future research with larger sample sizes is needed. A larger sample size is also necessary to examine the incidence of depressive and anxious symptomatology, by excluding those patients with depressive and anxiety symptoms at baseline. The sample size of this study was too small, unfortunately.

## Conclusion

Our study highlights the importance of illness perceptions in the development of depressive and anxious symptomatology in patients with FMS. Developing treatment interventions aimed at modification of poor illness perceptions is therefore of interest. It has been shown that interventions focusing on illness perceptions, such as cognitive treatment and illness perception focused intervention, is effective in improving maladaptive perceptions in patients with chronic low back pain (58), and even has a positive effect on depressive and anxious symptomatology (59). In addition, cognitive behavior therapy focusing on catastrophizing has shown to be effective at increasing acceptance of the illness and global functioning in patients with FMS (60). Our findings corroborate the recommendations of the EULAR for psychological treatment, such as cognitive behavior therapy, in case of depressed mood (15). Thus, strengthening illness beliefs and reducing catastrophic thinking seem to be important factors in the treatment of patients with FMS, allowing them to improve their health. Future research should further explore the effects of such interventions.

## Ethics Statement

This study was carried out in accordance with the recommendations of the local medical ethical board with written informed consent from all subjects. All subjects gave written informed consent in accordance with the Declaration of Helsinki. The Institutional Review Board of the University Medical Centre, Nijmegen, approved the study (protocol number: 2011/271).

## Author Contributions

EH, CF-C, and JV conceived the initial idea for this study, and all the authors contributed to its planning, including defining the aims, variables of interest, and analysis strategy. Analyses were done by JV, but all the authors had access to the statistical outputs. EH drafted the article, and all the authors contributed to revisions., approved the final manuscript, and agreed to be accountable for all aspects of the work in ensuring that questions related to the accuracy or integrity of any part of the work are appropriately investigated and resolved.

## Conflict of Interest Statement

The authors declare that the research was conducted in the absence of any commercial or financial relationships that could be construed as a potential conflict of interest.

## References

[1] Wolfe F Clauw DJ Fitzcharles MA Goldenberg DL Hauser W Katz RS Fibromyalgia criteria and severity scales for clinical and epidemiological studies: a modification of the ACR preliminary diagnostic criteria for fibromyalgia. J Rheumatol (2011) 38:6. 10.3899/jrheum.100594 21285161

[2] Chang MH Hsu JW Huang KL Su TP Bai YM Li CT Bidirectional association between depression and fibromyalgia syndrome: a nationwide longitudinal study. J Pain (2015) 16(9):895–902. 10.1016/j.jpain.2015.06.004 26117813

[3] Jay GW Barkin RL Fibromyalgia. Dis Mon (2015) 61:66–111. 10.1016/j.disamonth.2015.01.002 25769552

[4] Aguglia A Salvi V Maina G Rossetto I Aguglia E. Fibromyalgia syndrome and depressive symptoms: comorbidity and clinical correlates. J Affect Disord (2011) 128:262–6. 10.1016/j.jad.2010.07.004 20674985

[5] Gracely RH Ceko M Bushnell MC Review article: fibromyalgia and depression. Pain Res Treat (2012) 2012:486590 10.1155/2012/486590 22191023 PMC3236322

[6] Janssens KAM Zijlema WL Joustra ML Rosmalen JGM. Mood and anxiety disorders in chronic fatigue syndrome, fibromyalgia, and irritable bowel syndrome: results from the lifelines cohort study. Psychosom Med (2015) 77:449–57. 10.1097/PSY.0000000000000161 25768845

[7] Kayhan F Küçük A Satan Y İlgün E Arslan S İlik F Sexual dysfunction, mood, anxiety, and personality disorders in female patients with fibromyalgia. Neuropsychiatr Dis Treat (2016) 12:349–55. 10.2147/NDT.S99160 26937190 PMC4762461

[8] Sancassiani F Machado S Ruggiero V Cacace E Carmassi C Gesi C The management of fibromyalgia from a psychosomatic perspective: an overview. Int Rev Psychiatry (2017) 29:473–88. 10.1080/09540261.2017.1320982 28681628

[9] Ramiro Fde S Lombardi Júnior I da Silva RCB Montesano FT de Oliveira NRC Diniz REAS Investigation of stress, anxiety and depression in women with fibromyalgia: a comparative study. Rev Bras Reumatol (2013) 54(1):27–32. 10.1016/j.rbre.2014.02.003 24878788

[10] Bair MJ Robinson RL Katon W Kroenke K. Depression and pain comorbidity: a literature review. Arch Intern Med (2003) 163:2433–45. 10.1001/archinte.163.20.2433 14609780

[11] Gustavsson A Svensson M Jacobi F Allgulander C Alonso J Beghi E Cost of disorders of the brain in Europe 2010. Eur Neuropsychopharmacol (2001) 21:718–79. 10.1016/j.euroneuro.2011.08.008 21924589

[12] Kawai K Kawai AT Wollan P Yawn BP Adverse impacts of chronic pain on health-related quality of life, work productivity, depression and anxiety in a community-based study. Fam Pract (2017) cmx034:1–6. 10.1093/fampra/cmx034 PMC6260800 28444208

[13] Wittchen HU Jacobi F Rehm J Gustavsson A Svensson M Jonsson B The size and burden of mental disorders and other disorders of the brain in Europe 2010. Eur Neuropsychopharmacol (2011) 21:655–79. 10.1016/j.euroneuro.2011.07.018 21896369

[14] Carville SF Arendt-Nielsen S Bliddal H Blotman F Branco JC Buskila D EULAR evidence-based recommendations for the management of fibromyalgia syndrome. Ann Rheum Dis (2008) 67(4):536–41. 10.1136/ard.2007.071522 17644548

[15] Macfarlane GJ Kronisch C Dean LE Atzeni F Häuser W Fluß E EULAR revised recommendations for the management of fibromyalgia. Ann Rheum Dis (2016) 76(2):318–28. 10.1136/annrheumdis-2016-209724 27377815

[16] Vink D Aartsen MJ Schoevers RA. Risk factors for anxiety and depression in the elderly: a review. J Affect Disord (2008) 106(1):29–44. 10.1016/j.jad.2007.06.005 17707515

[17] Gureje O Von Korff M Kola L Demyttenaere K He Y Posada-Villa J The relation between multiple pains and mental disorders: results from the World Mental Health surveys. Pain (2008) 135:82–91. 10.1016/j.pain.2007.05.005 17570586

[18] Ligthart L Visscher CM van Houtem CMHH Geels LM Vink JM de Jongh A Comorbidity among multiple pain symptoms and anxious depression in a Dutch population sample. J Pain (2014) 15(9):945–55. 10.1016/j.jpain.2014.06.007 24981129

[19] Gerrits MMJG van Oppen P van Marwijk HWJ Penninx BW van der Horst HE Pain and the onset of depressive and anxiety disorders. Pain (2014) 155:53–9. 10.1016/j.pain.2013.09.005 24012953

[20] Gerrits MMJG van Oppen P Leone SS van Marwijk HWJ van der Horst HE Penninx BW. Pain, not chronic disease, is associated with the recurrence of depressive and anxiety disorders. BMC Psychiatry (2014) 14:187. 10.1186/1471-244X-14-187 24965597 PMC4090396

[21] Nicassio PM Radojevic V Schoenfeld-Smith K Dwyer K. The contribution of family cohesion and the pain-coping process to depressive symptoms in fibromyalgia. Ann Behav Med (1995) 17(4):349–56. 10.1007/BF02888600 24203602

[22] Kwakkenbos L van Lankveld WGJM Vonk MC Becker ES van den Hoogen FHJ van den Ende CHM. Disease-related and psychosocial factors associated with depressive symptoms in patients with systemic sclerosis, including fear of progression and appearance self-esteem. J Psychosom Res (2012) 72(3):199–204. 10.1016/j.jpsychores.2011.12.005 22325699

[23] Smith CA Wallston KA Dwyer KA Dowdy SW Beyond good and bad coping – a multidimensional examination of coping with pain in persons with rheumatoid arthritis. Ann Behav Med (1997) 19(1):11–21. 10.1007/BF02883422 9603673

[24] Costa ECV Vale S Sobral M Pereira MG Illness perceptions are the main predictors of depression and anxiety symptoms in patients with chronic pain. Psychol Health Med (2016) 21(4):483–95. 10.1080/13548506.2015.1109673 26560801

[25] Järemo P Arman M Gerdle B Larsson B Gottberg K Illness beliefs among patients with chronic widespread pain – associations with selfreported health status, anxiety and depressive symptoms and impact of pain. BMC Psychol (2017) 5:24 10.1186/s40359-017-0192-1 28679446 PMC5499007

[26] Sharpe L Sensky T Allard S. The course of depression in recent onset rheumatoid arthritis: the predictive role of disability, illness perceptions, pain and coping. J Psychosom Res (2001) 51:713–9. 10.1016/S0022-3999(01)00266-5 11750293

[27] Hassett AL Cone JD Patella SJ Sigal LH. The role of catastrophizing in the pain and depression of women with fibromyalgia syndrome. Arthritis Rheum (2000) 43(11):2493–500. 10.1002/1529-0131(200011)43:11<2493::AID-ANR17>3.0.CO;2-W 11083273

[28] Mellegård M Grossi G Soares JJ A comparative study of coping among women with fibromyalgia, neck/shoulder and back pain. Int J Behav Med (2001) 8(2):103–15. 10.1207/S15327558IJBM0802_02

[29] Nahlen Bose C Elfström ML Björling G Persson H Saboonchi F. Patterns and the mediating role of avoidant coping style and illness perception on anxiety and depression in patients with chronic heart failure. Scand J Caring Sci (2016) 30:704–13. 10.1111/scs.12297 26766405

[30] Le Grande MR Elliott PC Worcester MUC Murphy BM Goble AJ Kugathasan V Identifying illness perception schemata and their association with depression and quality of life in cardiac patients. Psychol Health Med (2012) 17(6):709–22. 10.1080/13548506.2012.661865 22416847

[31] Wolfe F Smythe HA Yunus MB Bennett RM Bombardier C Goldenberg DL The American College of Rheumatology 1990 criteria for the classification of fibromyalgia. Report of the Multicenter Criteria Committee. Arthritis Rheum (1990) 33:160–72. 10.1002/art.1780330203 2306288

[32] Wolfe F Clauw DJ Fitzcharles MA Goldenberg DL Katz RS Mease P The American College of Rheumatology preliminary diagnostic criteria for fibromyalgia and measurement of symptom severity. Arthritis Care Res (Hoboken) (2010) 62(5):600–10. 10.1002/acr.20140 20461783

[33] Zigmond AS Snaith RP. The hospital anxiety and depression scale. Acta Psychiatr Scand (1983) 67:361–70. 10.1111/j.1600-0447.1983.tb09716.x 6880820

[34] Herrmann C International experiences with the hospital anxiety and depression scale: a review of validation data and clinical results. J Psychosom Res (1997) 42(1):17–41. 10.1016/S0022-3999(96)00216-4 9055211

[35] Herrmann C Brand DS Kaminsky B Leibing E Staats H Ruger U. Diagnostic groups and depressed mood as predictors of 22-month mortality in medical inpatients. Psychosom Med (1998) 60(5):570–7. 10.1097/00006842-199809000-00011 9773760

[36] Mayou RA Gill D Thompson DR Day A Hicks N Volmink J Depression and anxiety as predictors of outcome after myocardial infarction. Psychosom Med (2000) 62(2):212–9. 10.1097/00006842-200003000-00011 10772399

[37] Bjelland I Dahl AA Haug TT Neckelmann D The validity of the hospital anxiety and depression scale: an updated literature review. J Psychosom Res (2002) 52(2):69–77. 10.1016/S0022-3999(01)00296-3 11832252

[38] Burckhardt CS Clark SR Bennett RM. The fibromyalgia impact questionnaire: development and validation. J Rheumatol (1991) 18:728–33. 1865419

[39] Zijlstra TR Taal E van de Laar MA Rasker JJ. Validation of a Dutch translation of the fibromyalgia impact questionnaire. Rheumatology (2007) 46(1):131–4. 10.1093/rheumatology/kel171 16757485

[40] Kraaimaat FW Bakker A Evers AWM Pijncoping-strategieën bij chronische pijnpatiënten: De ontwikkeling van de Pijn Coping Inventarisatielijst (PCI). (Pain coping strategies in chronic pain patients: the development of the Pain Coping Inventory (PCI)). Gedragstherapie (1997) 30(3):185–201.

[41] Kraaimaat FW Evers AW. Pain-coping strategies in chronic pain patients: psychometric characteristics of the Pain-Coping Inventory (PCI). Int J Behav Med (2003) 10(4):343–63. 10.1207/S15327558IJBM1004_5 14734263

[42] Moss-Morris R Weinman J Petrie KJ Horne R Cameron LD Buick D The revised illness perception questionnaire (IPQ-R). Psychol Health (2002) 17(1):1–16. 10.1080/08870440290001494

[43] van Ittersum MW van Wilgen CP Hilberdink WKHA Groothoff JW van der Schans CP Illness perceptions in patients with fibromyalgia. Patient Educ Couns (2009) 74:53–60. 10.1016/j.pec.2008.07.041 18815004

[44] Nicholls EE Hill S Foster NE. Musculoskeletal pain illness perceptions: factor structure of the illness perceptions questionnaire-revised. Psychol Health (2013) 28(1):84–102. 10.1080/08870446.2012.714782 22891727 PMC3516816

[45] Burcusa SL Iacono WG. Risk for recurrence in depression. Clin Psychol Rev (2007) 27(8):959–85. 10.1016/j.cpr.2007.02.005 17448579 PMC2169519

[46] Rezaei F Doost HTN Molavi H Abedi MR Karimifar M Depression and pain in patients with rheumatoid arthritis: mediating role of illness perception. Egypt Rheumatol (2014) 36:57–64. 10.1016/j.ejr.2013.12.007

[47] Bhagwagar Z Cowen PJ ‘It’s not over when it’s over’: persistent neurobiological abnormalities in recovered depressed patients. Psychol Med (2008) 38:307–13. 10.1017/S0033291707001250 18444278

[48] Borsook D Sava S Becerra L. The pain imaging revolution: advancing pain into the 21st century. Neuroscientist (2010) 16:171–85. 10.1177/1073858409349902 20400714 PMC3370428

[49] van Vliet IM de Beurs E The MINI-international neuropsychiatric interview. A brief structured diagnostic psychiatric interview for DSM-IV and ICD-10 psychiatric disorders. Tijdschr Psychiatr (2007) 49(6):393–7. 17614093

[50] Kessler RC Üstün TB The World Mental Health (WMH) survey initiative version of the World Health Organization (WHO) Composite International Diagnostic Interview (CIDI). Int J Methods Psychiatr Res (2004) 13(2):93–121. 10.1002/mpr.168 15297906 PMC6878592

[51] Anderson E Shivakumar G Effects of exercise and physical activity on anxiety. Front Psychiatry (2013) 4:27 10.3389/fpsyt.2013.00027 23630504 PMC3632802

[52] Blake H Physical activity and exercise in the treatment of depression. Front Psychiatry (2012) 3:106 10.3389/fpsyt.2012.00106 23233842 PMC3516810

[53] Stanton R Reaburn P. Exercise and the treatment of depression: a review of the exercise program variables. Med Sci Sports Sci (2014) 17(2):177–82. 10.1016/j.jsams.2013.03.010 23602562

[54] Stonerock GL Hoffman BM Smith PJ Blumenthal JA. Exercise as treatment for anxiety: systematic review and analysis. Ann Behav Med (2015) 49(4):542–56. 10.1007/s12160-014-9685-9 25697132 PMC4498975

[55] Krysta K Krzystanek M Janas-Kozik M Klasik A Krupka-Matuszczyk I. Impact of pharmacological and psychological treatment methods of depressive and anxiety disorders on cognitive functioning. J Neural Transm (2015) 122(1):101–10. 10.1007/s00702-014-1282-3 25078256 PMC4529445

[56] Kang HJ Kim SY Bae KY Kim SW Shin IS Yoon JS Comorbidity of depression with physical disorders: research and clinical implications. Chonnam Med J (2015) 51:8–18. 10.4068/cmj.2015.51.1.8 25914875 PMC4406996

[57] Lilympaki I Makri A Vlantousi K Koutelekos I Babatsikou F Polikandrioti M. Effect of perceived social support on the levels of anxiety and depression of hemodialysis patients. Mater Sociomed (2016) 28(5):361–5. 10.5455/msm.2016.28.361-365 27999485 PMC5149439

[58] Siemonsma PC Stuive I Roorda LD Vollebregt JA Walker MF Lankhorst GJ Cognitive treatment of illness perceptions in patients with chronic low back pain: a randomized controlled trial. Phys Ther (2013) 93(4):435–48. 10.2522/ptj.20110150 23162040

[59] Sararoudi RB Motmaen M Maracy MR Pishghadam E Kheirabadi GR. Efficacy of illness perception focused intervention on quality of life, anxiety, and depression in patients with myocardial infarction. J Res Med Sci (2016) 21:125. 10.4103/1735-1995.196607 28331511 PMC5348829

[60] Alda M Luciano JV Andrés E Serrano-Blanco A Rodero B del Hoyo YL Effectiveness of cognitive behaviour therapy for the treatment of catastrophisation in patients with fibromyalgia: a randomised controlled trial. Arthritis Res Ther (2011) 13:R173 10.1186/ar3496 22018333 PMC3308108

